# Avo-AirDB: An avocado UAV Database for agricultural image segmentation and classification

**DOI:** 10.1016/j.dib.2022.108738

**Published:** 2022-11-09

**Authors:** Khalid EL Amraoui, Mouataz Lghoul, Ayoub Ezzaki, Lhoussaine Masmoudi, Majid Hadri, Hicham Elbelrhiti, Aziz Abdou Simo

**Affiliations:** a LCS Laboratory, Physics Dept. faculty of science, Mohammed 5 university in Rabat, 4 ibn Battouta Street, Rabat, Morocco; b Department of Fundamental and Applied Sciences (DSFA), Hassan II Agronomic and Veterinary Institute, BP 6202 Madinat Al Irfan, Rabat, Morocco; c ABAZ company, Allal tazi, Kenitra, Morocco

**Keywords:** Dataset, Avocado, Unmanned aerial vehicles, Digital agriculture

## Abstract

Unmanned aerial vehicles (UAVs) with on-board cameras have the advantage of providing Bird-view images (Aerial images). This type of image is considered as a rich source of information especially for intelligent agriculture. A dataset of 984 aerial images of avocado threes is made publicly available with a ground resolution of 2.7 cm per pixel. It has been collected from over 113 Hectares of an avocado farm in ALLAL TAZI region of Morocco using a DJI Phantom 4 Pro UAV. It comprises original bird view and annotated images. The dataset is available at https://data.mendeley.com/datasets/tvhh83r3hj/2

**Specifications Table**

**Table:** 

Subject:	Applied Machine Learning
Specific subject area:	Artificial intelligence, Computer Vision and Digital Agriculture
Type of data:	RGB Bird-view images
How the data were acquired:	Unmanned Aerial Vehicle DJI Phantom Pro 4: • Max H-Speed:72 Km/h • Max V-speed:35 km/h • Weight 1.5 Kg • Action radius 500 m • Autonomy 20-30 Min • Radio control Frequency 2.4 GHz RGB Camera: • Sensor CMOS • Lens: 8.8 mm/24 mm • FOV: 84° • Resolution: 5472 × 3648 • Supported formats: JPG, PNG and RAW • Operating Temperature Range: 0° to 40°C
Data format:	Raw and analyzed
Description of data collection:	The dataset images were collected using the described UAV over a 113 Ha farm of Avocado trees. The camera angle was adjusted to 90° vertically with the field. The speed and flight altitude were 9 m/s and 90 meters respectively. A 75% Longitudinal overlap was applied.
Data source location:	• City/Town/Region: Kenitra/Allal Tazi Region • Country: Morocco • GPS Coordinates of the avocado farm: 34°34′55.4″N 6°21′60.0″W
Data accessibility	Repository name: Avo-AirDB Direct URL to the data: https://data.mendeley.com/datasets/tvhh83r3hj/2 DOI: 10.17632/tvhh83r3hj.2 Dataset description: https://github.com/LCSkhalid/Avo-AirDB

## Value of the Data

• The dataset represents a significant contribution to different applications in the digital agriculture field, such as: trees segmentation, trees counting and classification based on tree's crown surface, disease detection, etc.
• The data can be used by artificial intelligent (AI) researchers in addition to agricultural researches and professionals.
• The dataset is suitable for developing digital and precision agricultural systems.
• Collected data can be employed to train Artificial intelligence methods for image classification.
• The presented data is the only public dataset of Avocado aerial high-resolution images in the African continent.

## 1 Objective

The progress of agricultural visual pattern recognition (especially for avocado) one of the fundamental aspects of human beings, has been relatively slow [1]. Due to the low number of countries with an avocado production of more than 50K tonnes per year (only 22 countries), what is causing a lack of relevant datasets to encourage the study of agricultural images of avocado and visual patterns with many unique characteristics. The objective of this Dataset is to encourage research on this challenging task.

## 2 Data Description

**Fig. 1:**
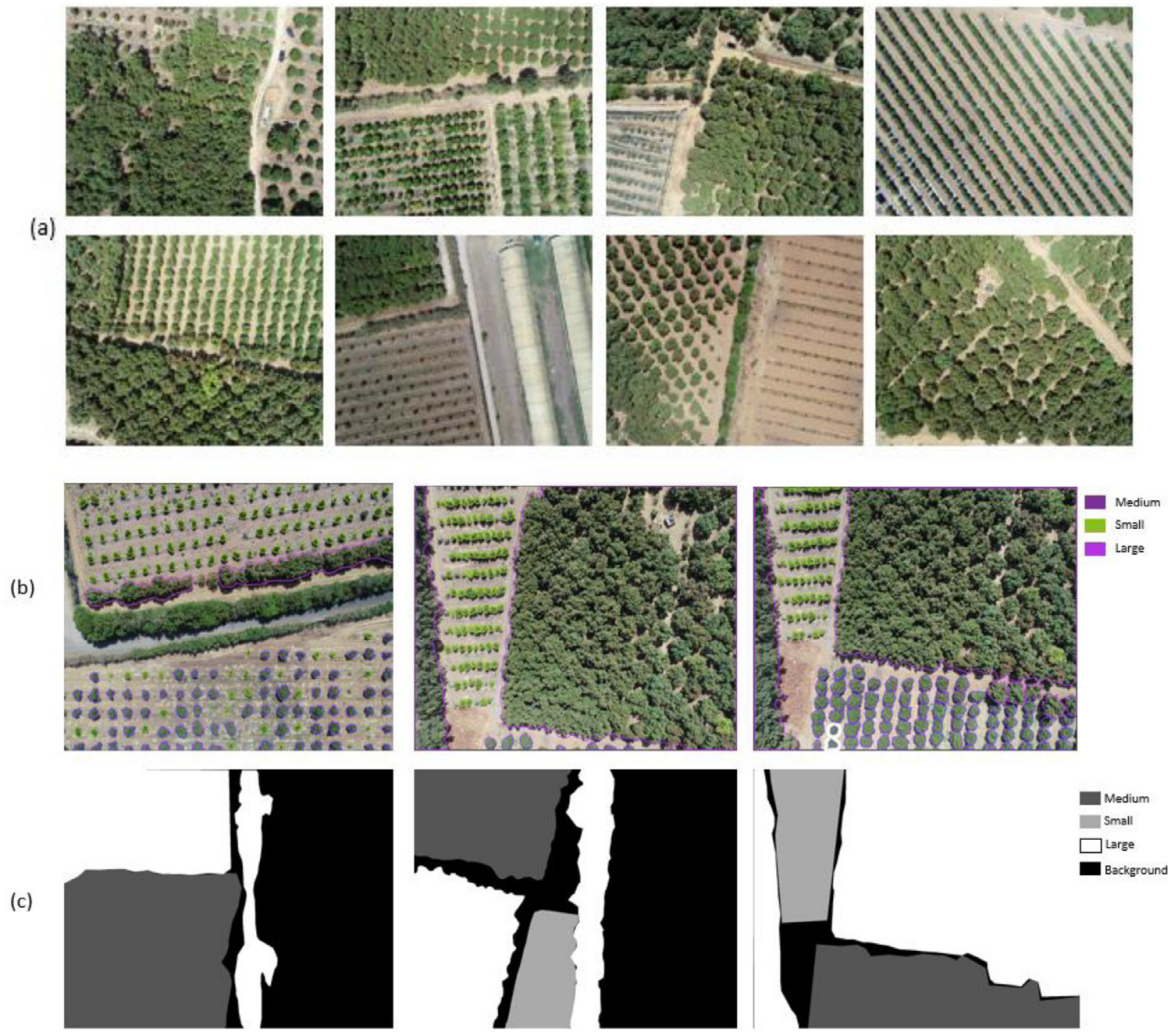
Sample images of the dataset. (a) RGB Aerial images. (b) Example of annotated images by make-sens.ai. (c) Example of annotated images by apeer.com.

**Fig. 2:**
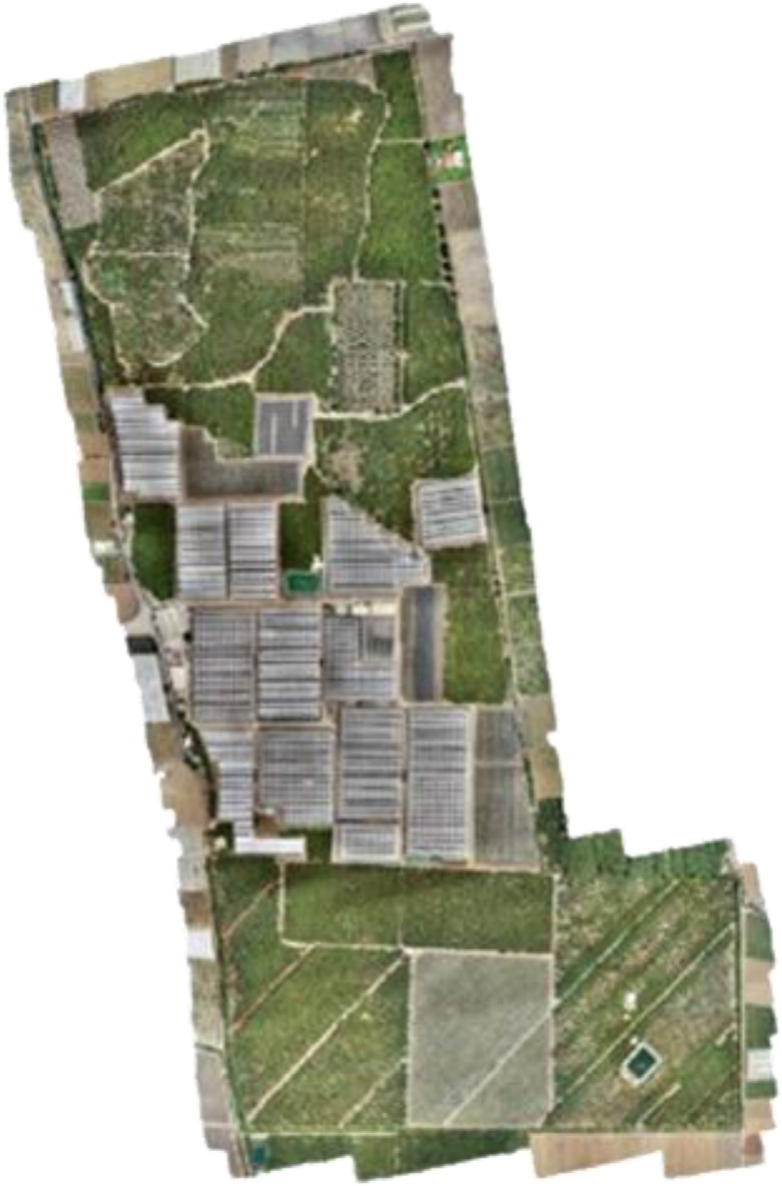
Visible orthoimage.

This paper describes a dataset of images collected by an unmanned aerial vehicle (UAV) from an avocado farm of more than 113 Ha leading to a set of 984 RGB images of 4864×3648 pixels. 93 images were annotated using the Make-sens.ai [2] and Apeer.com [3] platforms, forming Four classes, namely: Small, Medium, Large and background. In Fig. 1 samples from the developed dataset are provided while the Table 1 presents the folders and files organization of the dataset, and Table 2 and Table 3 represents the specifications of the used UAV and camera respectively. Fig. 2 shows the visible orthoimage using the Agisoft Metashape Software [4]

**Table 1:** Dataset organization.

Folder	Filename	Description
Avo-AirDB	DJI_XXXX.JPG	RGB aerial images numbered as XXXX (0002-0987)
annotation1/Images	DJI_XXXX.JPG	RGB aerial images used for creating the Masks numbered as XXXX (0002 -0057)
annotation1/Masks	DJI_XXXX.TIFF	Masks images numbered as XXXX (0002 -0057)
annotation2/Images	DJI_XXXX.JPG	RGB aerial images used for annotation numbered as XXXX (between 0002-0987)
annotation2/labels	VGG_Label.json	Contain images labels
	Readme.md	A text file containing the Dataset description and instructions to the users

**Table 2:** Specifications of the used UAV.

DJI PHONTOM 4 PRO					
Max H-Speed	Max V-speed	Weight	Action radius	Autonomy	Radio control Frequency
72 Km/h	35 km/h	1.5 Kg	500 m	20-30 Min	2.4 GHz

**Table 3:** Technical characteristics of the used camera.

FC6310 Camera					
Sensor	Lens	FOV	Max Resolution	Supported formats	Operating Temperature Range
CMOS	8.8 mm/24 mm	84°	5472 × 3648	2.61 × 2.61 µm	0° to 40°C

## 3 Experimental Design, Materials and Methods

### 3.1 UAV flight mission and data acquisition

Flight planning ultimately helps achieve mission objectives, keep flight altitude restrictions in mind, avoid restricted airspace, and improve battery life performance. For cartography, it can be very helpful to plan the number of flight paths or waypoints, the time required to complete the flight mission, the area to be flown, the number of images taken in a given area and the overlap between the pictures. In addition, different parameters should be taken into account:

**Atmospheric conditions**: Aerial survey missions are considered successful only if you obtain image quality that serves the scope of the planned mission. Weather conditions such as severe weather, crosswinds, or the wrong season can greatly affect the results. The position of the sun is a major factor in creating shadows. Generally, the best time to take aerial photos is between 10:00 am and 2:00 pm. Depending on the latitude of our scan area, around 12 o'clock is the best time due to the smallest shadows.

**Weather**: The weather is an essential parameter during the execution of the mission. For that, a forecast condition check in real-time is important. Table 4 gives the weather conditions during the mission time.

**Table 4:** Weather conditions during the acquisition mission.

Relative humidity %	Weather condition	Temperature°C	Wind Speed km/h
73	Partially clear	19-27	6-19

**Planification**: The mission planification necessity the setting up of different parameters for an optimal mission control. For our mission, we used DroneDeploy which is one of the most used and sophisticated Drone mapping software [5]. Table 5 presents the parameters of the acquisition mission.

**Table 5:** Parameters of the acquisition mission.

Flight altitude	90 m
Estimated resolution	2.7 cm/px
Longitudinal overlap	75%
Lateral overlap	65%
Flight direction	168%
Speed	9 m/s
Camera angle	90°
Surface	113 Ha
Flight time	50 min
Number Of batteries	4

**Fig. 3:**
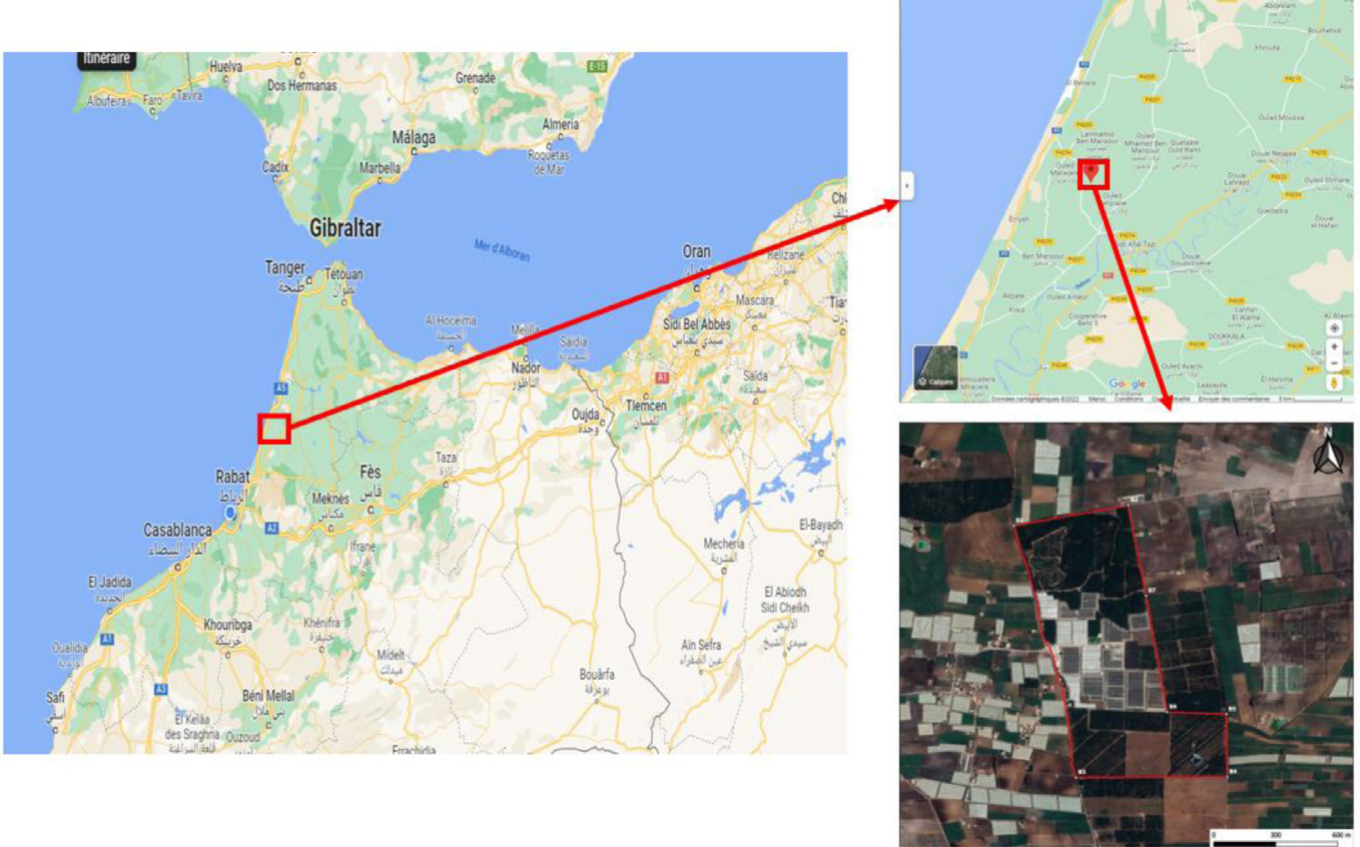
Dataset acquisition region.

**Fig. 4:**
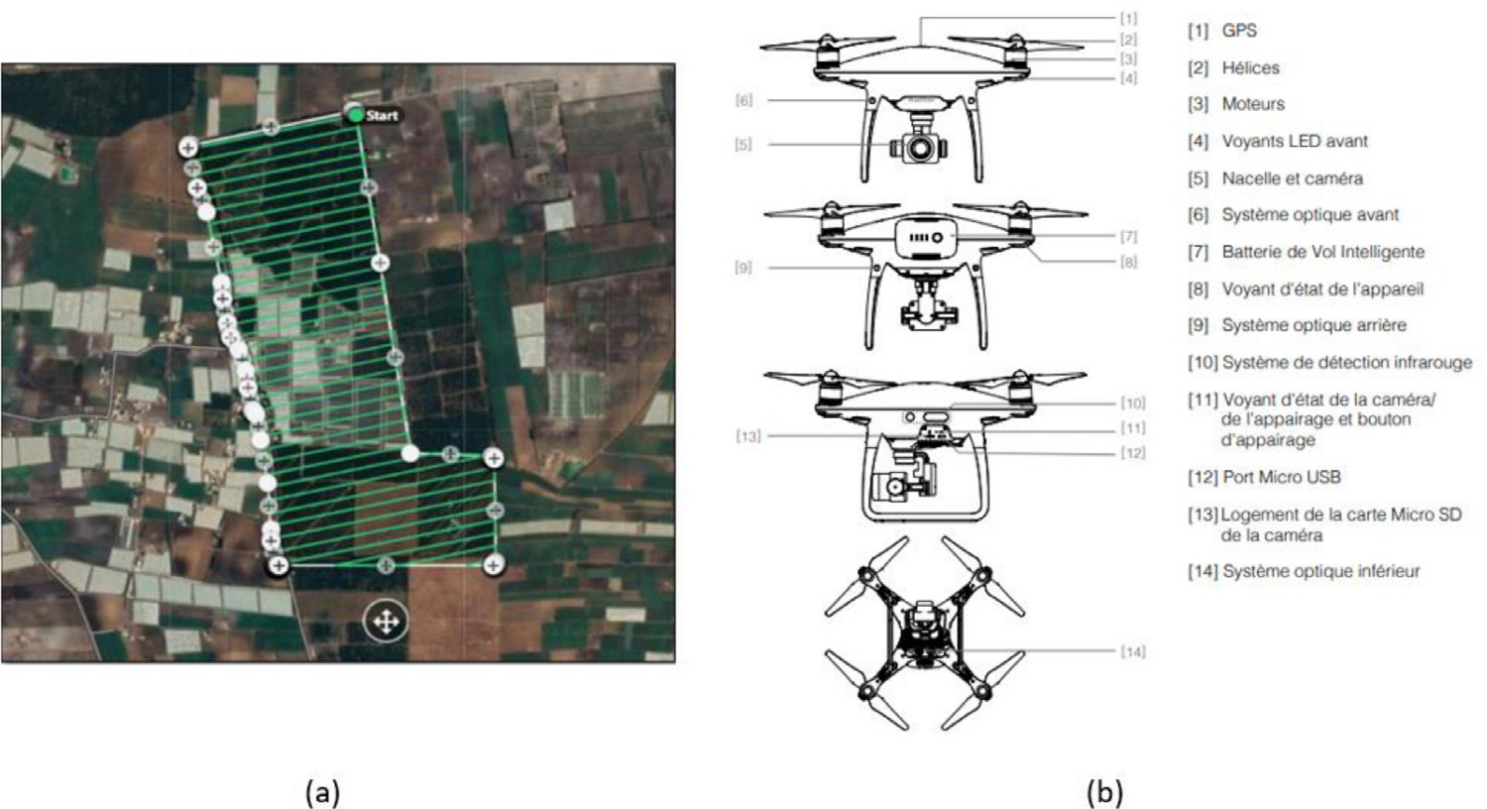
(a) The flight path of the acquisition mission by the used UAV. (b) Different components of the used DJI Phantom 2 Pro.

The data collection was carried out using the described UAV (Fig. 4.b) and based on the navigation scheme presented in Fig. 4.a, provided by Drone-deploy system in order to cover the entire avocado farm (Fig. 3) and guaranteeing a maximum mission efficiency.

### 3.2 Data annotation

The collected data were manually annotated by our agricultural experts in the avocado field using two online platforms Apeer and makeSense. Images were annotated using a polygon outline, leading to Four classes in the first annotation (by Apeer platform): Small, Medium, Large regions and the Background, and to three classes in the second annotation (by Make-sense platform): Small, Medium and Large trees. The aims are to propose annotated images that can be used in different machine learning applications. Table 6 summarized the statistics of the most related datasets of agriculture images. Seg refers to segmentation and Cls is refers to classification.

**Table 6:** diffrents datasets for agricultural images.

Dataset	Images	Classes	Labels	Tasks	Image size (Pixels)	Channels	Resolution (GSD)
Crop discrimination [6]	60	2	494	Seg.	1296 × 966	RGB	N/A
Senseftly Crop Field [7]	5260	N/A	N/A	N/A	N/A	NRG, Red Edge	12.13cm/px
DeepWeeds [8]	17509	1	17509	Cls.	1920 × 1200	RGB	N/A
Agriculture-Vision [9]	94986	9	169086	Seg.	512 × 512	RGB, NIR	10/15/20 cm/px
**Avo-AirDB**	**984**	**3/4**	**93**	**Cls**	**4864 × 3648**	**RGB**	**2.7cm/px**

## Ethics Statements

The dataset does not include animal in experiments, human subjects or data collected from social media.

## CRediT Author Statement

**Khalid EL Amraoui**: Methodology, Software, Data curation and validation. **Moataz Lghoul**: Software and Data curation. **Ayoub Ezzaki**: Validation, Investigation, Data curation and Writing – Original Draft. **Lhoussaine Masmoudi**: Conceptualization, Supervision, Project administration, funding acquisition and Writing - Review & Editing. **Majid Hadri**: Resources and Investigation. **Hicham El Belrhiti**: Conceptualization and Writing - Review & Editing. **Aziz Abdou Simo**: Resources.

## Declaration of Competing Interest

The authors declare that they have no known competing financial interests or personal relationships that could have appeared to influence the work reported in this paper.

## Data Availability

Avo-AirDB: An avocado UAV Database for agricultural image segmentation and classification (Original data) (Mendeley Data)
